# The Nucleosome Remodelling and Deacetylation complex coordinates the transcriptional response to lineage commitment in pluripotent cells

**DOI:** 10.1242/bio.060101

**Published:** 2024-01-22

**Authors:** Bertille Montibus, Ramy Ragheb, Evangelia Diamanti, Sara-Jane Dunn, Nicola Reynolds, Brian Hendrich

**Affiliations:** ^1^ Wellcome – MRC Stem Cell Institute, University of Cambridge, Cambridge CB2 0AW, United Kingdom; ^2^ Department of Haematology, University of Cambridge, Cambridge CB2 0AW, UK; ^3^ Microsoft Research, 21 Station Road, Cambridge CB1 2FB, UK; ^4^ Department of Biochemistry, University of Cambridge, Cambridge CB2 1QR, UK

**Keywords:** Chromatin, Embryonic stem cell, Lineage commitment, MBD3, NuRD, Transcription

## Abstract

As cells exit the pluripotent state and begin to commit to a specific lineage they must activate genes appropriate for that lineage while silencing genes associated with pluripotency and preventing activation of lineage-inappropriate genes. The Nucleosome Remodelling and Deacetylation (NuRD) complex is essential for pluripotent cells to successfully undergo lineage commitment. NuRD controls nucleosome density at regulatory sequences to facilitate transcriptional responses, and also has been shown to prevent unscheduled transcription (transcriptional noise) in undifferentiated pluripotent cells. How these activities combine to ensure cells engage a gene expression program suitable for successful lineage commitment has not been determined. Here, we show that NuRD is not required to silence all genes. Rather, it restricts expression of genes primed for activation upon exit from the pluripotent state, but maintains them in a transcriptionally permissive state in self-renewing conditions, which facilitates their subsequent activation upon exit from naïve pluripotency. We further show that NuRD coordinates gene expression changes, which acts to maintain a barrier between different stable states. Thus NuRD-mediated chromatin remodelling serves multiple functions, including reducing transcriptional noise, priming genes for activation and coordinating the transcriptional response to facilitate lineage commitment.

## Teaser

**Summary:** We show that the chromatin remodeller NuRD suppresses transcriptional noise, primes genes for activation and coordinates the transcriptional response in individual cells during the exit from naive pluripotency.

## INTRODUCTION

Cells in multicellular organisms arise from a single zygote and, with very few exceptions, inherit the same DNA content through cell divisions. Cells in the preimplantation mammalian embryo are totipotent, meaning that they can give rise to all embryonic and extraembryonic cell types of the developing organism. As development progresses, cells lose potency as they transit through different states to finally acquire an identity associated with their function. The transitions between different states are tightly regulated, which ensures that the correct number of cells is provided for each cell type. This fate acquisition is accompanied by the establishment of an expression profile with specific genes expressed and others actively repressed.

Embryonic stem cells (ES cells) are a powerful system in which to identify differentiation signals involved in cell state transitions. They are highly stable when maintained in the naïve state, yet have the potential to form all cell types in an adult organism once allowed to leave that state (Wray et al., 2010; Ying et al., 2008). Indeed, the autocrine signals which drive ES cells to exit pluripotency and initiate lineage commitment, and the cellular machinery important for cells to properly interpret and respond to these signals, have been studied extensively (Gökbuget and Blelloch, 2019; Kalkan et al., 2019; Lackner et al., 2021; Leeb et al., 2014; Yang et al., 2012). Integration of external cues by cells leads to changes in the gene regulatory network that defines cell state, and changes in those cues can further modify the stability of different cell states as development proceeds (Signolet and Hendrich, 2015).

Gene expression is controlled to a large extent by how the relevant regulatory sequences – enhancers and promoters – are packaged in chromatin. Chromatin density is largely controlled by protein complexes containing an ATP-dependent catalytic subunit which can move, evict, recruit or assemble nucleosomes (Hota and Bruneau, 2016; Narlikar et al., 2013). One such remodeller that is known to facilitate cell fate decisions is the Nucleosome Remodelling and Deacetylase (NuRD) complex (Ahringer, 2000). NuRD contains both chromatin remodelling and lysine deacetylase activities in distinct subcomplexes (Allen et al., 2013; Low et al., 2020). These two subcomplexes are held together by the MBD2 or MBD3 proteins, which themselves are mutually exclusive in NuRD complexes (Burgold et al., 2019; Guezennec et al., 2006). In the absence of an MBD2/3 protein, the complex falls apart, which does not immediately displace the remodelling component, called CHD4, but does alter its activity (Bornelöv et al., 2018). MBD3/NuRD is required for successful lineage commitment of pluripotent cells both in culture and *in vivo* (Kaji et al., 2006, 2007). NuRD components localise to sites of active transcription in multiple cell types, binding to both enhancers and promoters (Bornelöv et al., 2018; Günther et al., 2013; Mathieu et al., 2012; Shimbo et al., 2013). Rather than simply activating or repressing transcription, NuRD functions to both dampen unscheduled transcription in the undifferentiated state, and to modulate active transcription as cells are undergoing lineage commitment. Together, these transcriptional regulatory activities allow cells to respond appropriately to inductive signals, facilitating lineage commitment (Bornelöv et al., 2018; Burgold et al., 2019; Ragheb et al., 2020).

While it is clear that NuRD activity is essential for successful lineage commitment of pluripotent cells, exactly how its transcriptional regulatory activity facilitates lineage commitment is not clear. NuRD activity suppresses inappropriate transcription, or transcriptional noise, in both human and mouse pluripotent cell cultures, but it is not known how this activity is read out in individual cells, nor how cells respond when this noise reduction activity is compromised. Here we used single cell RNAseq across the first 48 h of ES cell exit from the naïve state to define how Mbd3/NuRD activity functions to facilitate lineage commitment. We determined how NuRD activity impacts the ability of cells to control gene expression as they exit the self-renewing state and identified a function for NuRD in ensuring that cells progress in a coordinated fashion along the early developmental trajectory. This analysis clarifies the role of an important and abundant chromatin remodeller in control of gene expression during lineage commitment, which we show is significantly more nuanced than the now-outdated model of remodellers simply switching genes on or off.

## RESULTS

### MBD3/NuRD activity is required for mouse ES cells to express lineage-appropriate genes

**Fig. 1.**
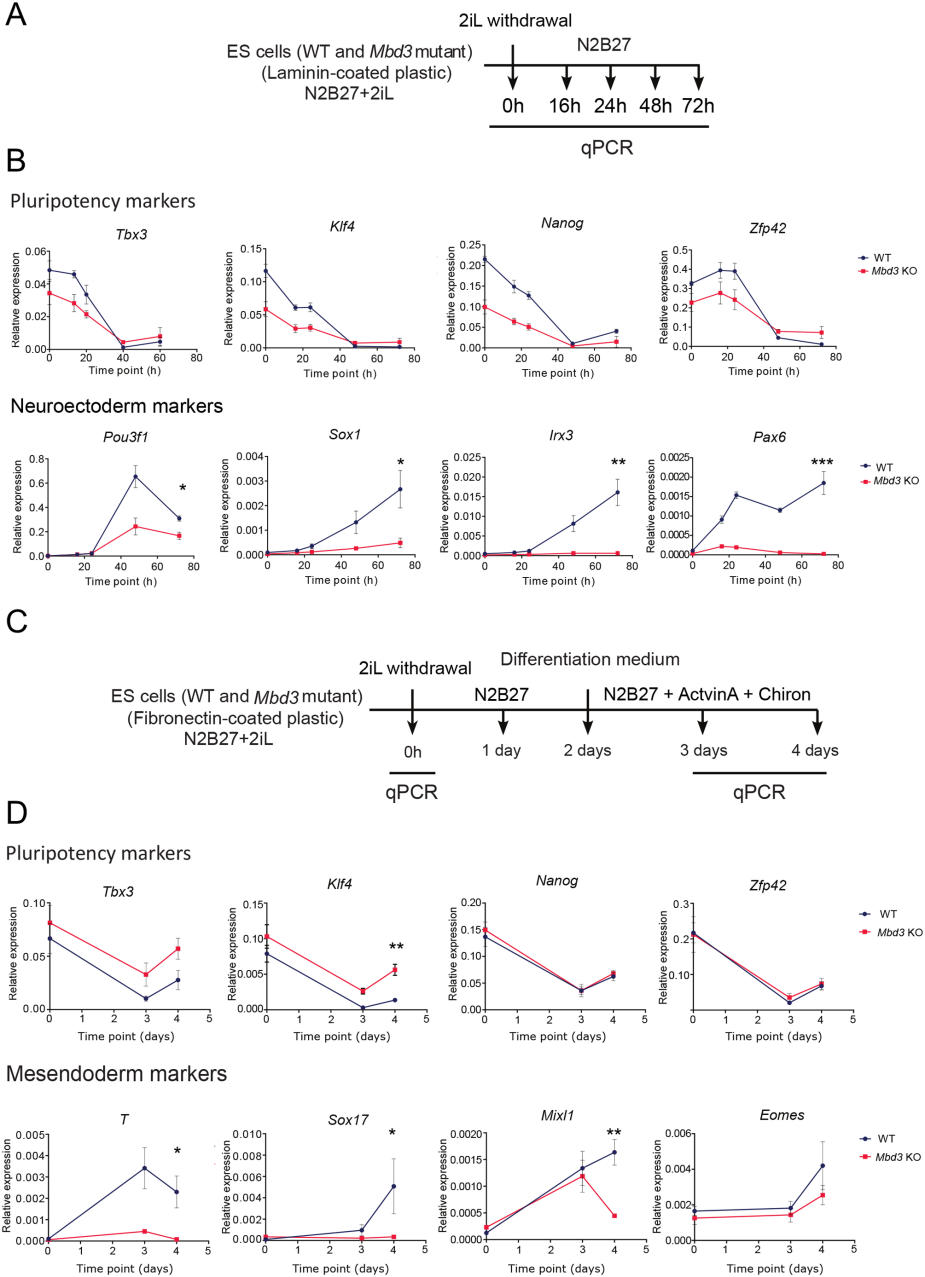
Gene expression analysis at the population level confirms the defect of induction of lineage genes during differentiation in *Mbd3* mutant cells. (A) Protocol for monolayer neuroectodermal differentiation of naïve ES cells in N2B27 (Kalkan et al., 2017). (B) Gene expression analysis by RT-qPCR at the population level for selected representative pluripotency markers and neuroectoderm markers during neuroectoderm differentiation. Expression was normalised to three housekeeping genes (*Gapdh*, *Atp5A1*, *Ppia*). Error bars indicate the standard error of four independent differentiations. (C) Protocol for mesendoderm differentiation of naïve ES cells in N2B27 supplemented with Activin A and Chiron (Turner et al., 2014). (D) Gene expression analysis by RT-qPCR at the population level for selected representative pluripotency markers and mesendoderm markers during mesendoderm differentiation. Error bars indicate the standard error of four independent differentiations. Asterisks in B and D indicate that wild type and mutant are significantly different at the final time point by two-tailed *t*-test (*P<0.05, **P<0.01, ***P<0.001).

To define the average behaviour of Mbd3/NuRD mutant ES cells during the early stages of lineage commitment, we investigated gene expression changes by RT-qPCR, which occur as cells leave the naïve state and begin differentiating down the neuroectodermal lineage. In wild-type (WT) cells, pluripotency-associated genes (*Tbx3*, *Klf4*, *Nanog* and *Zfp42*) were rapidly downregulated, while neuroectoderm markers (*Pou3f1*, *Sox1*, *Irx3* and *Pax6*) were activated steadily across a 72 h differentiation time course (Fig. 1A). By contrast, while *Mbd3* mutant cells downregulated the pluripotency-associated genes, they failed to induce the expression of lineage genes by 72 h (Fig. 1B). This same result was seen in two independently derived *Mbd3*-null ES cell lines (Fig. S1). To determine whether the defect in the induction of the expression of lineage genes was limited to neuroectoderm genes, we conducted a similar analysis during mesendoderm differentiation (Fig. 1C). Here again, the *Mbd3* mutant cells failed to properly induce the expression of lineage genes after 4 days (*T*, *Sox17*, *Mixl1*), despite being able to downregulate expression of pluripotency genes (Fig. 1D). These results are consistent with our previous results differentiating ES cells or primed human and mouse pluripotent cells lacking NuRD activity (Burgold et al., 2019; Ragheb et al., 2020), and indicate that a fully functional NuRD complex is required for cells to respond properly to differentiation signals and induce expression of lineage-specific genes.

### NuRD facilitates a robust and uniform transcriptional response to differentiation cues

**Fig. 2.**
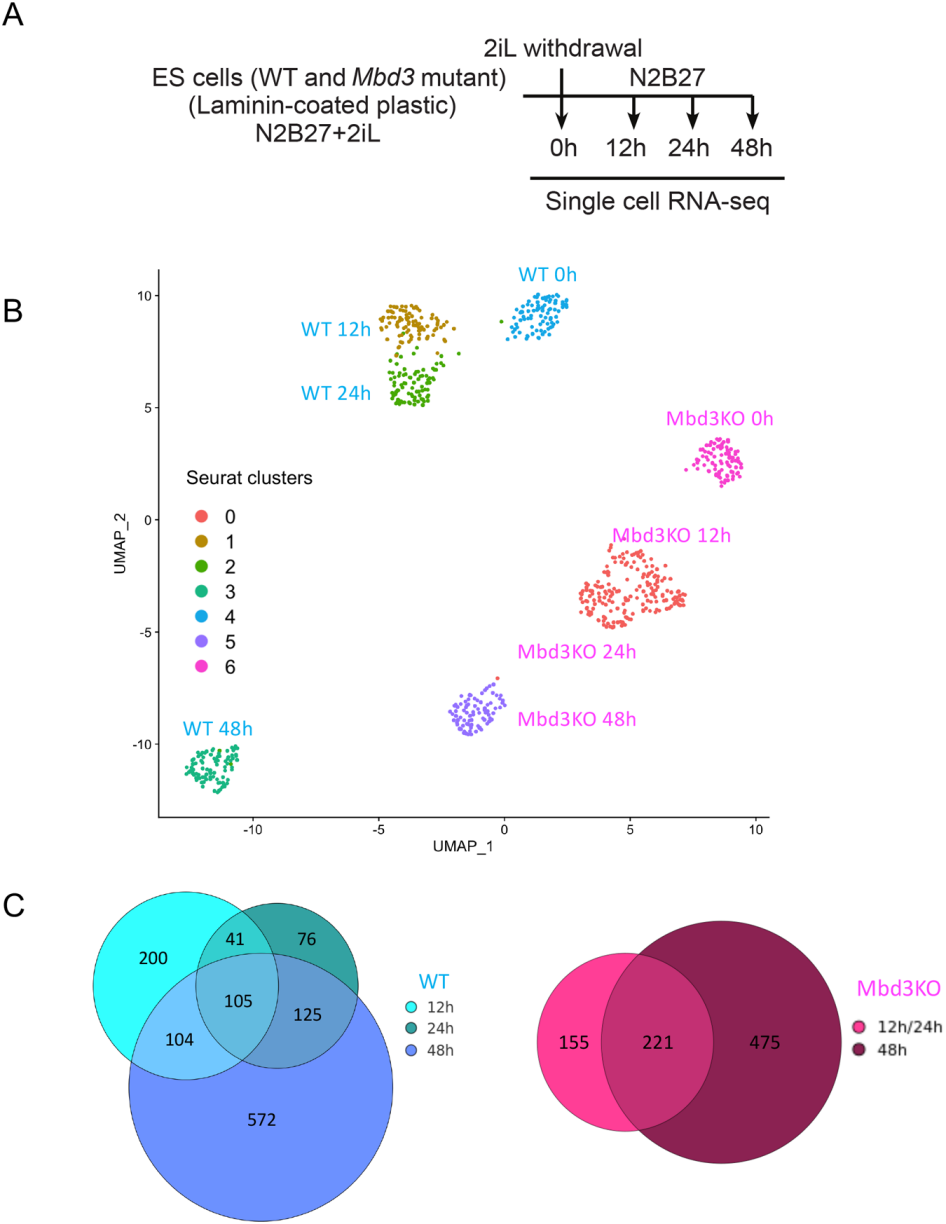
**Single cell gene expression analysis of exit from the naïve state in wild type and *Mbd3*-knockout ES cells.** (A) Scheme of differentiation time course for the single cell RNA sequencing dataset. (B) UMAP representation of the single cell RNAseq data using all genes. Only cells that have passed quality control are plotted. Cells are coloured according to their Seurat cluster affiliation and labelled with their biological identities. (C) The numbers of significantly (*P*<0.05) differentially expressed genes in each Seurat clusters (reassigned to their corresponding biological condition) are shown for wild type (left) and *Mbd3*-knockout cells (right).

NuRD activity is required to suppress transcriptional noise as pluripotent cells initiate lineage commitment, and cells lacking NuRD activity can respond to differentiation cues but cannot establish the correct gene regulatory networks required for successful lineage commitment (Burgold et al., 2019; Ragheb et al., 2020; Signolet and Hendrich, 2015). To explore the role of NuRD in control of gene expression during lineage commitment in more detail, we assayed mRNA expression in individual wild-type or *Mbd3*-null ES cells in self-renewing conditions (2iL) and at 12, 24 and 48 h after removal of both inhibitors and LIF (Fig. 2A). We chose to use SMRT-seq2 rather than a more high-throughput method as we wished to maximise the depth of mRNA recovery to better detect lowly expressed genes. We therefore sequenced 96 cells per time point and per genotype, making a total of 768 cells, 739 of which passed quality control and were used for subsequent analyses (Fig. S2A).

Visualising the data as a UMAP representation shows that wild-type and mutant cells cluster separately throughout the differentiation time course (Fig. 2B). Despite clustering separately at the 2iL time point, both wild-type and mutant cells show movement through the UMAP plot across the differentiation time course in the same general direction (Fig. 2B). Indeed, the expression of genes identified as early responders after 2iL withdrawal (Kalkan et al., 2017; Sokol, 2011) largely showed the expected direction of change in both wild-type and mutant cells (Fig. S2B). This indicates that NuRD activity is not required for cells to receive and begin to respond to differentiation cues. Yet the mutant cells only show limited movement across the UMAP plot (Fig. 2B). Further, while wild-type cells were separated into four clusters corresponding to differentiation time using the Seurat algorithm (see Materials and Methods), the same method could not bioinformatically distinguish between the 12 and 24 h time points for mutant cells (Fig. 2B).

Comparison of the number of differentially expressed genes (DEGs) at every time point showed an increase in gene expression changes over time in both genotypes (Fig. 2C). Enrichment analysis for gene ontologies on DEGs at 48 h indicated mostly general developmental terms and terms associated with metabolism and motility, with neural development being the predominant developmental lineage as expected when 2iL is removed from ES cell culture (Ying et al., 2003) (Table S2). Genes activated in mutant cells at 48 h were also associated with general developmental, metabolism and motility terms, although less significantly than wild type cells (Table S2).

**Fig. 3.**
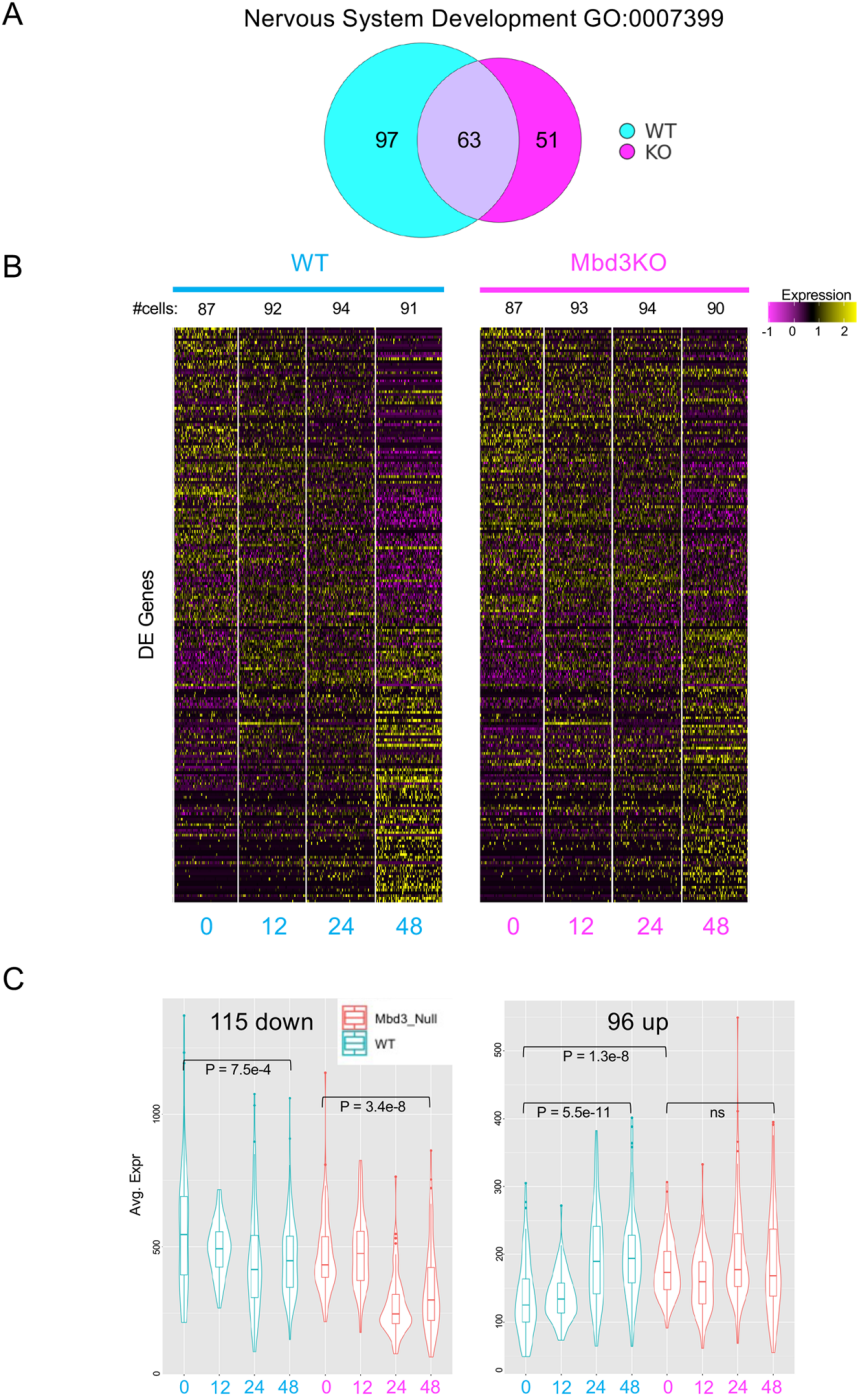
**Behaviour of 48 h differentially expressed Nervous System Development genes in wild type and mutant cells.** (A) Venn diagram showing the overlap of genes contained in the GO term “Nervous system development” (GO:0007399) which show significant expression changes after 48 h of differentiation in wild-type (blue) and *Mbd3*-knockout cells (magenta) (genes listed in Table S3). (B) Heatmap showing the log2 normalised expression level across the differentiation time course for genes described in A. The number of cells scored at each time point is indicated above. (C) Average expression profiles of gene sets shown in B divided into those decreasing in expression (*N*=114) or those increasing in expression (*N*=96). Wild-type expression patterns are shown in blue and expression in mutant cells shown in red. The shape of the violin indicates the proportion of cells at each position along the y-axis; the midline of the box shows the median value, the lower and upper edges of the box represent the 1st and the 3rd quartiles, and the whiskers represent the minimum and maximum values. *P*-values were calculated using a two-tailed *t*-test; ns, not significant.

The GO term “Nervous system development” was enriched for both the wild-type and the mutant differentially expressed genes after 48 h of differentiation (wild type: *P*=9.56E-06; knockout *P*=2.85E-02). We were surprised at this, given that *Mbd3*-null cells have an extremely low probability of successfully adopting a neuroectodermal fate, even in the correct embryonic context (Kaji et al., 2006). We therefore compared the behaviour of genes within this GO term between the wild type and mutant differentiation time courses (Fig. 3). Those genes within this group normally activated during differentiation showed inappropriately high expression in *Mbd3*-knockout cells in 2iL (Fig. 3C, right panel). These genes did not show a significant further increase in average expression across the time course in mutant cells. Surprisingly, more than half of the differentially expressed genes in this category are normally downregulated during exit from the naïve state, and this downregulation also occurs in the absence of Mbd3/NuRD (Fig. 3C, left panel). This is consistent with Mbd3/NuRD activity maintaining repression of lineage-associated genes, enabling them to be activated upon receipt of appropriate differentiation signals.

### Incomplete silencing of differentiation-associated genes precedes failure of their activation

**Fig. 4.**
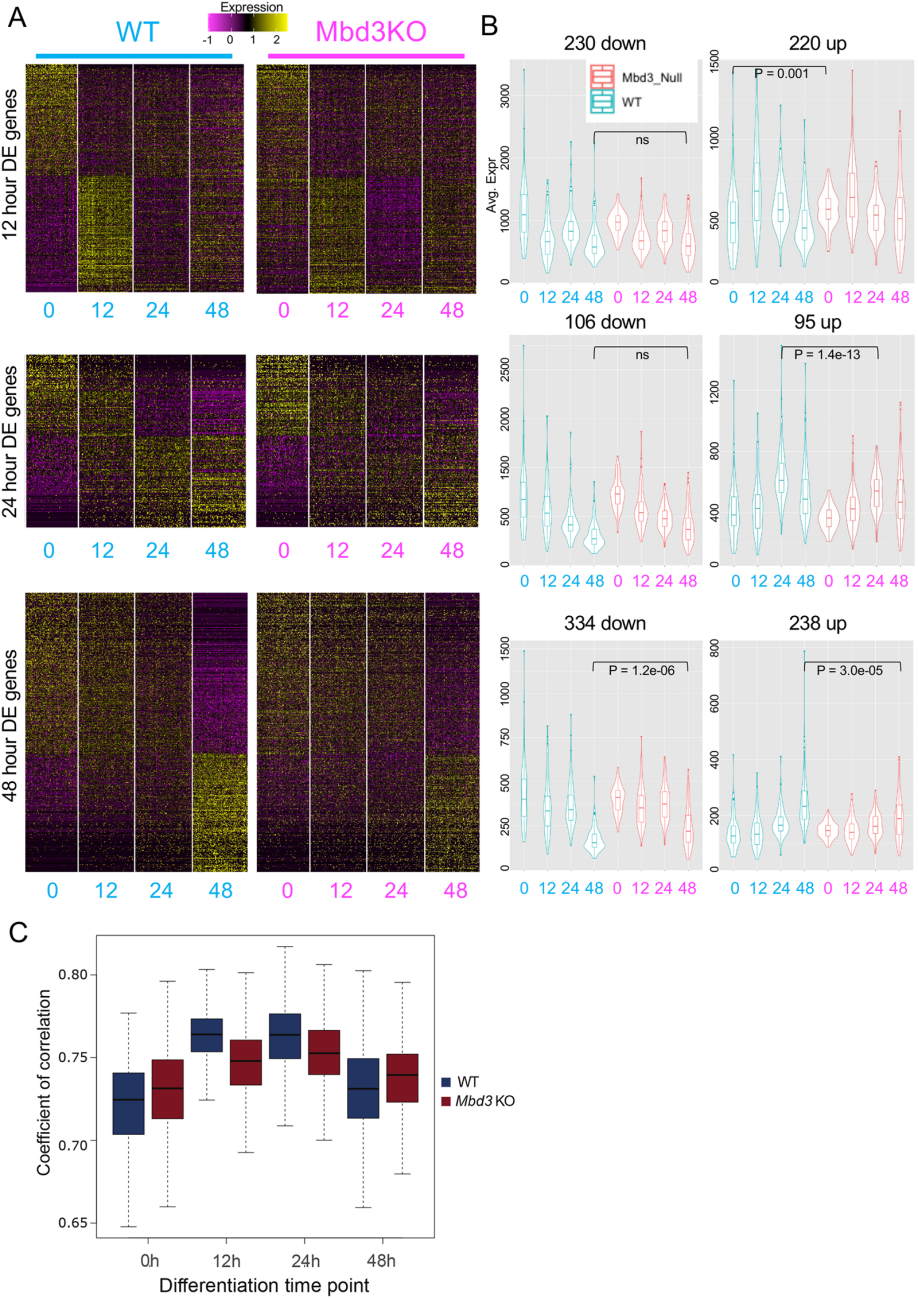
**NuRD activity is required both for noise reduction and for timely activation of specific gene subsets.** (A) Expression of genes significantly changing in wild-type cells is plotted across all time points, clustered according to fold change in wild-type cells. Expression of genes first showing significant changes at 12 h (top panels), 24 h (middle panels) or 48 h (bottom panels) are displayed for wild-type (left) and *Mbd3*-null cells (right). (B) Average expression profiles of gene sets shown in A, with wild type expression patterns shown in blue (left) and expression in mutant cells shown in red (right). The shape of the violin indicates the proportion of cells at each position along the y-axis; the midline of the box shows the median value, the lower and upper edges of the box represent the 1st and the 3rd quartiles, and the whiskers represent the minimum and maximum values. *P*-values were calculated using a two-tailed *t*-test; ns, not significant. (C) Distribution of the Pearson coefficient of correlation between the wild type (blue) and *Mbd3*-knockout cells (red) is shown at each time point of differentiation.

To define how NuRD-dependent gene regulation defects change over time, we identified those genes significantly changing expression in wild-type cells at each time point during the differentiation time course and compared how they behaved in mutant cells. Mbd3/NuRD was not generally required for gene downregulation, although those genes normally downregulated at 48 h in wild-type cells showed persistent expression in mutant cells (48 h DOWN genes, Fig. 4B, bottom left panel). The observed failure to completely repress transcription in *Mbd3*-null cells is consistent with the proposed function of Mbd3/NuRD to suppress transcriptional noise (i.e. widespread low-level failure of gene silencing) made using bulk RNA-seq data in both human and mouse pluripotent cells (Burgold et al., 2019; Ragheb et al., 2020).

Genes normally activated in wild-type cells at all three time points show increases in expression in *Mbd3*-null cells, but in all three cases the level of activation is reduced compared to that seen in wild-type cells. Those genes first activated at 12 h in wild-type cells show increased average expression in undifferentiated mutant cells (*P*_(WT0h versus KO0h)_=0.001; Fig. 4B, top panels) and a reduced degree of activation (average fold change wild type 0 h versus 12 h=1.56; average fold change knockout (KO) 0 h versus 12 h=1.09). Similarly, *Mbd3*-null cells showed reduced activation of those genes first upregulated in wild-type cells at 24 h (average fold change wild type 0 h versus 24 h=1.63; average fold change knockout 0 h versus 24 h=1.28; Fig. 4B middle panels) and 48 h (average fold change wild type 0 h versus 48 h=1.56; average fold change knockout 0 h versus 48 h=1.12; Fig. 4B bottom panels). Together these data indicate that NuRD activity is important both for transcriptional activation as cells exit the naïve state and for complete repression of genes normally silenced as cells transit through formative pluripotency.

It has been reported that embryonic cells undergo a decrease in transcriptional heterogeneity (i.e. variation in gene expression among cells) as they exit the naïve state, followed by an increase at the onset of lineage commitment (Mohammed et al., 2017). In other words, cells become more similar in terms of gene expression as they transition from the naïve state through the formative and primed states, and become more different again as they undergo lineage commitment. To determine whether NuRD-deficient ES cells are capable of undergoing this transition, we calculated the pairwise correlation between cells of a given genotype and time point. Consistent with previous observations (Semrau et al., 2017), wild-type naïve ES cells initially undergo an increase in cell–cell correlation during the first 24 h of differentiation and experience a decrease in correlation as they begin to undergo lineage commitment at 48 h (Fig. 4C). *Mbd3*-null cells showed increased cell–cell correlation (*P*-value<2.2e-16; Welch's *t*-test) in self-renewing conditions compared to wild-type cells (Fig. 4C). Upon exit from the naïve state *Mbd3*-null cells showed a reduced extent of increased correlation by 24 h followed by a subsequent decrease at 48 h (Fig. 4C). Thus Mbd3/NuRD activity is not strictly required for the changes in transcriptional heterogeneity, which occur during exit from naïve pluripotency, but in its absence this change is muted compared to that seen in wild-type cells.

### Mbd3/NuRD maintains coordination of differentiation-induced gene expression changes

**Fig. 5.**
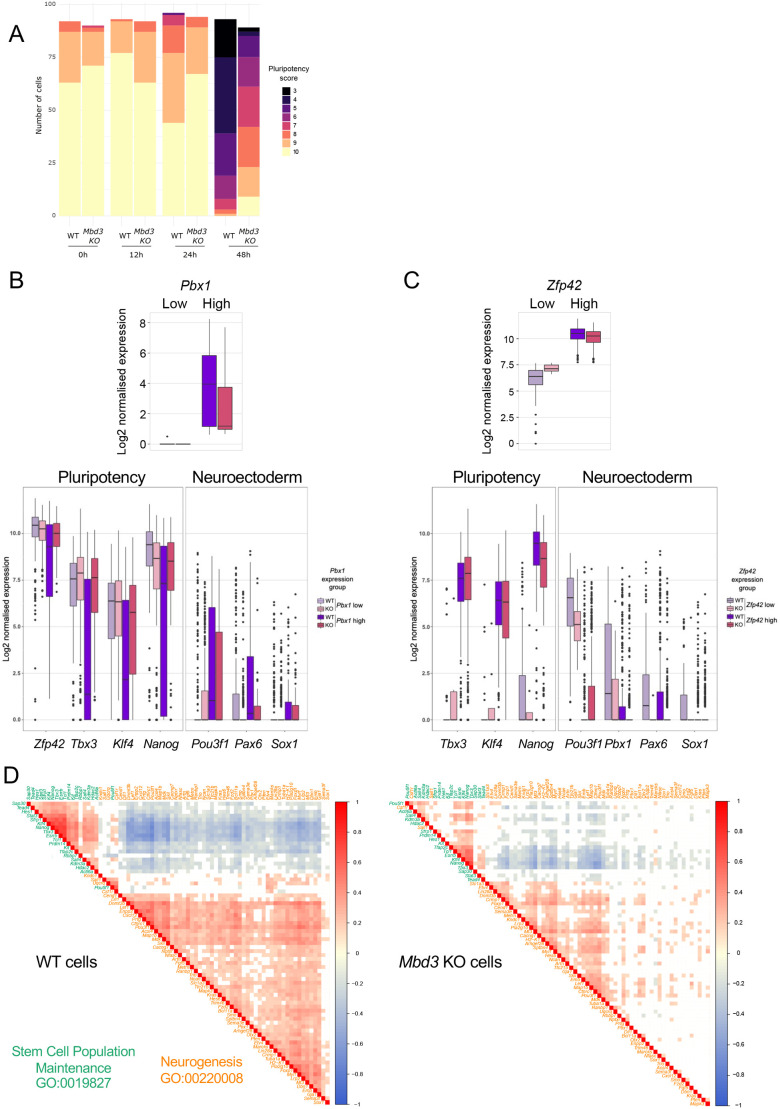
**NuRD is important for a coordinated transcriptional response to exit from the naïve state.** (A) Cells were discretised for expression of selected pluripotency-associated genes; that is, scored as 0 for low or off, and 1 for medium or high expression. These were then used to calculate a global score per cell. The cumulative number of cells for each pluripotency score is plotted for wild type and *Mbd3*-null cells at each time point. Colours indicate pluripotency scores. (B,C) Cells were classified as expressing “Low” or “High” levels of *Pbx1* (B) or *Zfp42* (C), top panels. Data for wild-type cells is shown in purple while those for *Mbd3*-knockout cells is shown in pink. Normalised expression of indicated markers of pluripotency or neuroectoderm was then plotted for wild-type (purple) and mutant (pink) cells classified as showing low or high (light or dark colours, respectively) expression of *Pbx1* (panel B) or *Zfp42* (panel C). (D) Pearson correlation analysis between the expression profiles of genes belonging to the Gene Omnibus (GO) terms “Stem Cell Population Maintenance” (GO:0019827) and “Neurogenesis” (GO:00220008) in wild-type cells (left panel) and in *Mbd3*-knockout cells (right panel). Data are shown for genes showing significant differential expression in the wild type differentiation time course and only the significant Pearson correlation coefficients were plotted. Lists of genes plotted in the order displayed in the figure are available in Table S4.

To get a picture of the behaviour of changes in the neural and pluripotency gene regulatory networks across the differentiation time course, we calculated a “fate score” per cell by classifying cells using k-means clustering into two groups according to their expression (high or low) for a set of representative pluripotency-associated genes (see Materials and Methods). Cells were discretised for expression of each selected gene; that is, a score of 1 was given to cells with high expression and 0 for cells with low expression. These scores were then used to calculate a global score per cell for groups of genes. Distribution of pluripotency scores among cells showed that, by 48 h, there were nine cells with the maximum pluripotency score (10) in the mutant cells, compared to none in the wild-type cells (Fig. 5A). Moreover, we observed higher heterogeneity in the distribution of the pluripotency score for the mutant cells after 48 h as compared to the wild-type cells which switch quickly from a high to low score after 24 h (Fig. 5A). This indicates that NuRD activity normally maintains the integrity of the gene regulatory network during differentiation, such that in the absence of Mbd3/NuRD cells variably deviate from the normal developmental trajectory leaving some able to initiate early developmental gene expression, some unable to leave the pluripotent state, and the rest at some point between these two extremes.

The decrease in expression of pluripotency genes but lack of, or reduced activation of, differentiation-associated markers in *Mbd3*-null ES cells after 48 h in differentiation conditions indicates that there is a lack of coordination of differentiation-associated gene expression changes in the absence of NuRD activity. To investigate this in more detail we firstly discretised expression levels for an early neural marker, *Pbx1* (Linares et al., 2015), as well as for a marker of naïve pluripotency, *Zfp42* (i.e. scored each as 1=high or 0=low/off). We classified cells as *Pbx1*-high (dark colours, Fig. 5B) or low (light colours) across all time points and plotted cells according to expression of selected pluripotency and neuroectoderm genes (Fig. 5B,C). In wild-type cells, expression of *Pbx1* is associated with reduced levels of pluripotency gene expression and activation of early differentiation genes, as expected for differentiating cells (dark purple boxplots, Fig. 5B). By contrast, in the mutant cells this coordination of gene expression changes is partially lost, with *Pbx1* high or low cells (dark or light pink, respectively; Fig. 5B) showing little difference in the expression of pluripotency markers. If we instead classify cells in terms of expression of *Zfp42*, a marker of naïve pluripotency, *Mbd3*-mutant *Zfp42*-low cells show both lower expression of neural markers and elevated expression of naïve markers *Tbx3* and *Klf4* as compared to wild-type *Zfp42*-low cells [compare light pink (Mbd3-knockout cells) with light purple (wild-type cells), Fig. 5C]. *Zfp42*-high cells (dark colours, Fig. 5C), which represent cells in the naïve state, show fewer differences in gene expression patterns. Together these data indicate a lack of coordination between silencing of the pluripotency gene expression network and activation of the neural differentiation programme in the absence of Mbd3/NuRD.

To obtain a broader view of the coordination of gene expression changes between the pluripotency and the neuroectoderm networks, we plotted correlation values between differentially expressed genes in wild-type cells contained in the GO terms “Stem Cell Population Maintenance” (GO:0019827) and “Neurogenesis” (GO:00220008) among cells at all time points for wild-type or mutant cells (Fig. 5D). Wild-type cells show the expected high correlations among genes within each GO term, but a strong anti-correlation between genes corresponding to different terms (Fig. 5D, left). In contrast, both the positive and negative correlations are much weaker in the *Mbd3*-knockout cells, indicating a failure of cells to adequately correlate the response to a shift out of the self-renewing state.

We therefore conclude that the essential function exerted by NuRD to facilitate lineage commitment is twofold: firstly, it reduces transcriptional noise globally in undifferentiated conditions, and secondly, it is required to mount an appropriate, coordinated transcriptional response to receipt of differentiation signals.

## DISCUSSION

Here we provide clarification of the essential role played by the NuRD complex in control of gene expression during ES cell lineage commitment. We find that NuRD is important both for keeping the early responding differentiation-associated genes repressed in the self-renewing state, and for their timely and efficient activation upon 2iL withdrawal. We suggest that this is because NuRD activity is required to render the regulatory sequences of these genes appropriately responsive to activation signals. It is notable that Mbd3/NuRD does not appear to be required for active downregulation of highly transcribed genes in ES cell differentiation, which must be achieved by some other repressive mechanism. Rather, our data support a model in which NuRD activity is necessary both to prevent precocious gene activation and to effectively prime genes for scheduled activation.

The ability to profile gene expression changes in individual cells allowed us to obtain a detailed picture of cellular behaviour during differentiation. While it is clear that NuRD-deficient cells are capable of downregulating components of the pluripotency gene regulatory network (Fig. 1) (Burgold et al., 2019; Ragheb et al., 2020), after 48 h of differentiation we can still identify some *Mbd3*-null cells with persistent expression of pluripotency markers (Fig. 5A). This explains why it was possible to recover self-renewing *Mbd3*-null ES cell colonies several days after differentiation induction (Reynolds et al., 2012). This indicates that the probability of completely inactivating the pluripotency gene regulatory network is reduced in the absence of Mbd3/NuRD activity. It also indicates that cells read this probability at least somewhat independently such that in a culture of millions of cells induced to differentiate, the vast majority will have silenced the pluripotency gene regulatory network but a small proportion have not. By contrast, NuRD activity in wild-type cells ensures that the probability of remaining pluripotent after exit from the self-renewing state is effectively zero. This difference, between a low probability and a near-zero probability, is important because persistence of even one pluripotent cell in a developing somatic lineage could result in teratoma formation.

NuRD proteins localise to regulatory regions of active genes in multiple cell types and CHD4-dependent nucleosome remodelling has been identified at both enhancers and promoters (Bornelöv et al., 2018; Dieuleveult et al., 2016; Günther et al., 2013; Liang et al., 2017; Mathieu et al., 2012; Ragheb et al., 2020; Shimbo et al., 2013). Enhancers are likely to be the important drivers of changes to gene expression during development (Gökbuget and Blelloch, 2019), so control of chromatin structure by NuRD at enhancers is likely to directly control their ability to respond to inductive signals. How chromatin remodellers and extracellular signals combine to alter enhancer chromatin has not been systematically investigated.

**Fig. 6.**
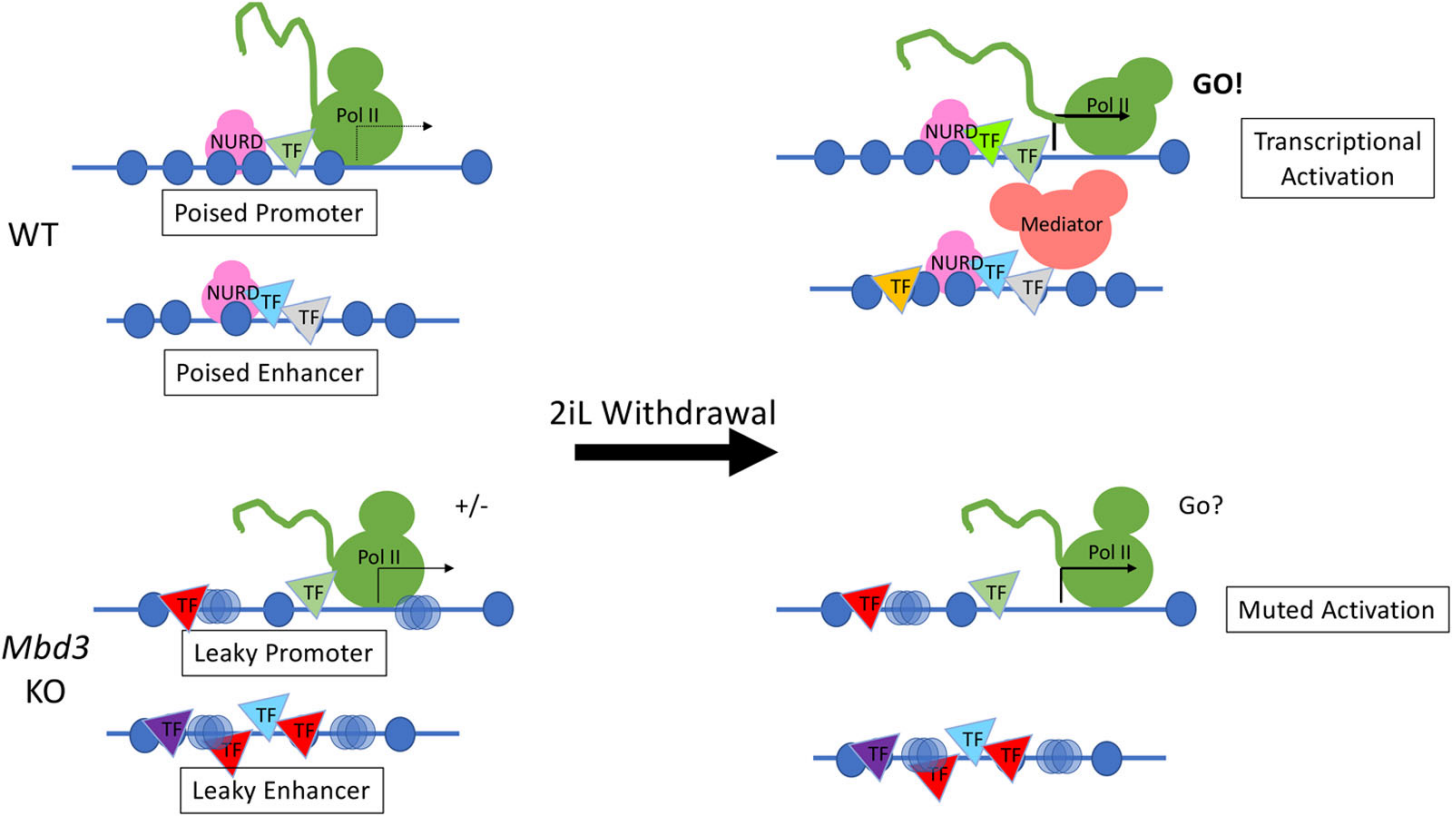
**Model of how NuRD facilitated transcriptional activation during differentiation.** In wild-type cells, NuRD activity ensures positioned nucleosomes (blue circles) and restricts transcription factor (triangles) binding at promoters and enhancers (top left). The promoter is poised, but inactive. The enhancers are very mobile in three-dimensional space, meaning they can scan a large area for sequences with which to interact. When cells are induced to differentiate (2iL withdrawal), NuRD maintains enhancer nucleosome structure and allows appropriate transcription factor binding so the enhancer and promoter can interact to drive differentiation-associated transcription (top right). In *Mbd3*-knockout cells, both promoters and enhancers have less positioned nucleosomes (hazy blue circles), inappropriate transcription factor binding (bottom left), and the enhancer is less mobile, meaning it can only sample its immediate vicinity. The lack of NuRD activity at the enhancer and/or promoter results in incomplete silencing (“+/-”). When induced to differentiate the enhancer is not in an appropriate chromatin configuration an cannot sample sufficient three-dimensional space to appropriately activate transcription. As a consequence, the promoter shows muted activation (bottom right).

In addition to controlling access of transcription factors and the general transcription machinery to regulatory sequences, Mbd3/NuRD also functions to increase the space explored by enhancers in the nucleus (Basu et al., 2023; Bornelöv et al., 2018). Whether these two activities are directly related remains to be determined. Nonetheless, we propose a scenario where enhancers of genes primed to respond to signals are held in an inactive but activatable state through NuRD-mediated chromatin remodelling (Fig. 6). Upon receipt of a differentiation signal these enhancers, which are in the correct chromatin configuration to rapidly become activated, can interact with relevant promoters and instruct gene expression changes. In the absence of NuRD, differentiation-associated enhancers are “leaky”, i.e. they are not maintained in a poised, but inactive state. Upon receipt of a differentiation signal, some responsive gene promoters become activated though the relevant enhancers, while others are unable to contact appropriate enhancers and hence are not activated appropriately. In the absence of a coordinated, robust transcriptional response lineage commitment is not possible.

Mbd3/NuRD-mutant ES cells are able to respond to signals that normally lead to lineage commitment upon 2iL withdrawal, however this response is not sufficiently robust to allow the cells to successfully enter the differentiation pathway. This phenotype is different from that seen in *Mbd3*-mutant somatic stem cells. For example, neural stem cells in the developing mouse cortex are able to undergo lineage commitment in the absence of Mbd3 to produce cortical projection neurons, but the neurons produced inappropriately express markers of multiple neural identities (Knock et al., 2015). In this case Mbd3/NuRD activity is dispensable for lineage commitment of a stem cell population but is required for fidelity of the cell types produced. In lymphocyte progenitors Mbd3/NuRD is important to restrict expression of dormant lineage gene expression programmes, maintaining an appropriate balance of pro-B cells and T cell progenitors produced during lineage commitment (Loughran et al., 2017). Different stem cell populations can thus use Mbd3/NuRD to control lineage commitment and differentiation to different extents. Similarly, mouse and human pluripotent stem cells both rely on NuRD activity for lineage commitment, but the details of how they use NuRD differs between cells in the two species (Ragheb et al., 2020). It is possible that seemingly different requirements for NuRD activity in different cell types could be due to tissue-specific differences in NuRD complex components (Reid et al., 2023). Alternately, and not mutually exclusively, it could be that NuRD exerts the same biochemical activity in all cell types, and it is the molecular architecture of the enhancers and promoters upon which NuRD acts that determines how that activity is used by the cell.

## MATERIALS AND METHODS

### Cell lines and differentiation

Mouse embryonic stem (ES) cells were grown on gelatin coated plates in N2B27 supplemented with recombinant mouse LIF (made in-house) and the inhibitors PD0325901 (abcr AB 253775) and CHIR99021 (abcr AB 253776) (2iL medium, made in-house) as described (Ying et al., 2008). Cell lines were genotyped and tested for mycoplasma contamination regularly. The cell lines used in this study are BHA-derived (wild type and *Mbd3*-null; 40, XX) (Stevens et al., 2017) for single cell RNAseq and the bulk of the molecular work and 7E12-derived wild type and *Mbd3*-null lines (40, XY) (Bornelöv et al., 2018) for independent verification of selected results.

Differentiation of embryonic stem cells toward neuroectoderm was induced by withdrawal of two inhibitors and LIF. The cells were grown on tissue culture laminin (Millipore CC095-5MG) coated plates during all differentiation. Differentiation of embryonic stem cells toward mesendoderm was performed on human-plasma-fibronectin (Millipore FC010) coated plates. Cells were grown for 2 days in N2B27 before adding 10 ng/ml recombinant human activin A (produced in-house) and 3 µM CHIR99021 in N2B27 to induce mesendoderm differentiation during subsequent days.

### Single cell RNA-sequencing

At each time point of the differentiation time course, cells were collected using accutase. The resulting cell suspension was sorted using a MoFlo cell sorter (Beckman Coulter) to deposit one cell per well of a 96-well plate in 2 μl of cell lysis buffer [0.2% (V/V) Triton-X100 (Sigma-Aldrich) and 2 U/µL of RNase inhibitor (Invitrogen 10777019)]. Library preparation was performed following the Smart-seq2 protocol (Picelli et al., 2014), and libraries were sequenced at the CRUK Cambridge Institute Genomics Core facility (Cambridge, UK) on an Illumina HiSeq4000 sequencer. The wild type scRNAseq datasets have also been analysed in (Lando et al., 2023 preprint).

### Bioinformatic analyses

RNA-seq libraries were aligned to the mouse reference genome (GRCm38/mm10) with the GSNAP aligner (gmap-2014-12-17) (Wu and Nacu, 2010) using the parameters -*n* 1 -*N* 1 for uniquely mapped reads and allowing for novel splicing sites. Gene read counts were assigned using HTSeq (v0.6.1) based on gene annotation from ensemble release 81. Quality control filtering was then applied to the cells using the R package Scater (1.2.0) (McCarthy et al., 2017): more that 5×10^5^ reads per cell, more than 7000 individual features detected and less than 5% of the reads mapping to mitochondrial genes. R package Seurat v.4.1.0 was used for cell filtration, normalization, principal component analysis, variable genes finding, clustering analysis, and uniform manifold approximation and projection (UMAP) dimensional reduction. The significantly differentially expressed genes were selected at an FDR-adjusted *P*-value equal or lower than 0.05. The gene ontology analysis was conducted using MouseMine (Motenko et al., 2015).

### Discretisation of the data, fate score and correlation analyses

The discretisation of the expression data for individual genes was conducted using k-means clustering into two groups except for the genes with unimodal distribution of expression. According to the clustering the cells were classified as low expressing cells or high expressing cells for each gene. The fate score for each cell was calculated using representative genes for every lineage and summing the number of genes with high expression for these genes. Representative pluripotency genes were *Pou5f1, Sall4, Zfp42, Klf2, Sox2, Tfcp2l1, Stat3, Nanog, Klf4,* and *Tbx3* (Dunn et al., 2014; Kalkan et al., 2017). The correlation analysis between gene expression profiles at all time points was performed for the set of genes corresponding to the GO terms “Stem Cell Population Maintenance” (GO:0019827) and “Neurogenesis” (GO:00220008), which were differentially expressed between 0 h and 48 h of differentiation in the wild-type cells (downregulated for GO:0019827 and upregulated for GO:00220008). The correlation analysis was conducted using a Pearson correlation test. Cell–cell correlation analysis was conducted by computing the Pearson coefficient of correlation between each pair of cells based on the gene expression of all the genes detected. This was done individually at each time point per genotype.

### Gene expression analysis

Total RNA was purified using Trizol reagent (Invitrogen) following the manufacturer instructions. DNase treatment was performed using DNase I and First-strand cDNA was synthesised using SuperScript IV reverse transcriptase (Invitrogen) with random hexamers. Quantitative PCRs (qPCRs) were performed using TaqMan reagents (Applied Biosystems) and SybrGreen (Applied Biosystems) on a QuantStudio Flex Real-Time PCR System (Applied Biosystems). Gene expression was determined relative to housekeeping genes (*Gapdh, Atp5A1, Ppia*) using the ΔCt method. Locus-specific primers used for quantitative PCR are listed in Table S1.

## Supplementary Material

10.1242/biolopen.060101_sup1 Supplementary information

Table S1.

Table S2.

Table S3.

Table S4.
