# Supplementary material for: The Nucleosome Remodelling and Deacetylation complex coordinates the transcriptional response to lineage commitment in pluripotent cells
Source: Biol Open. 2024 Jan 22;13(1):bio060101. doi: 10.1242/bio.060101 (PMC10836651; doi:10.1242/bio.060101)
Supplement: Supplementary information [file biolopen-13-060101-s1.pdf]

Biology Open (2023): doi:10.1242/bio.060101: Supplementary information

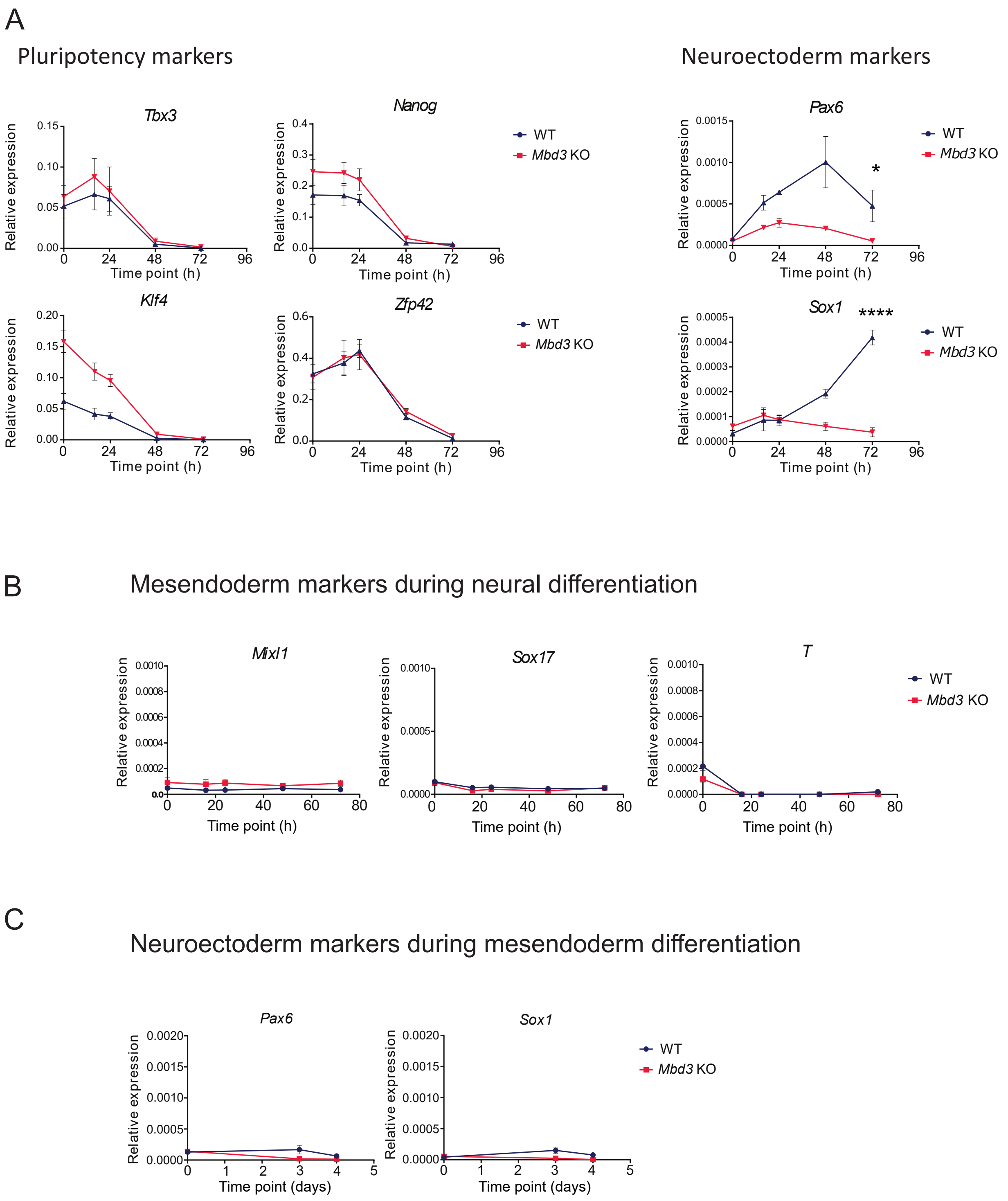

Fig. S1. *Mbd3* mutant cells show a defect of induction of lineage gene expression and an absence of other lineages activation during neuroectoderm differentiation. **A.** Gene expression analysis by RT-qPCR at the population level for selected representative pluripotency markers and neuroectoderm markers during neuroectoderm differentiation of another *Mbd3* KO cell line. Error bars indicate the standard error of 4 independent differentiations. Asterisks indicate wild type and mutant are significantly different at the final time point by two-tailed t-test (* $P<0.05$, **** $P<0.0001$). **B.** Gene expression analysis by RT-qPCR at the population level for selected representative mesendoderm markers during neuroectoderm differentiation. Error bars indicate the standard error of 4 independent differentiations. **C.** Gene expression analysis by RT-qPCR at the population level for selected representative neuroectoderm markers during mesendoderm differentiation. Error bars indicate the standard error of 4 independent differentiations.

A

Genotype	Differentiation time point	Number of cells sequenced	Number of cells kept after QC
WT	0h	96	92
	12h	96	93
	24h	96	96
	48h	96	93
Mbd3 KO	0h	96	90
	12h	96	92
	24h	96	94
	48h	96	89
Total		768	739

B

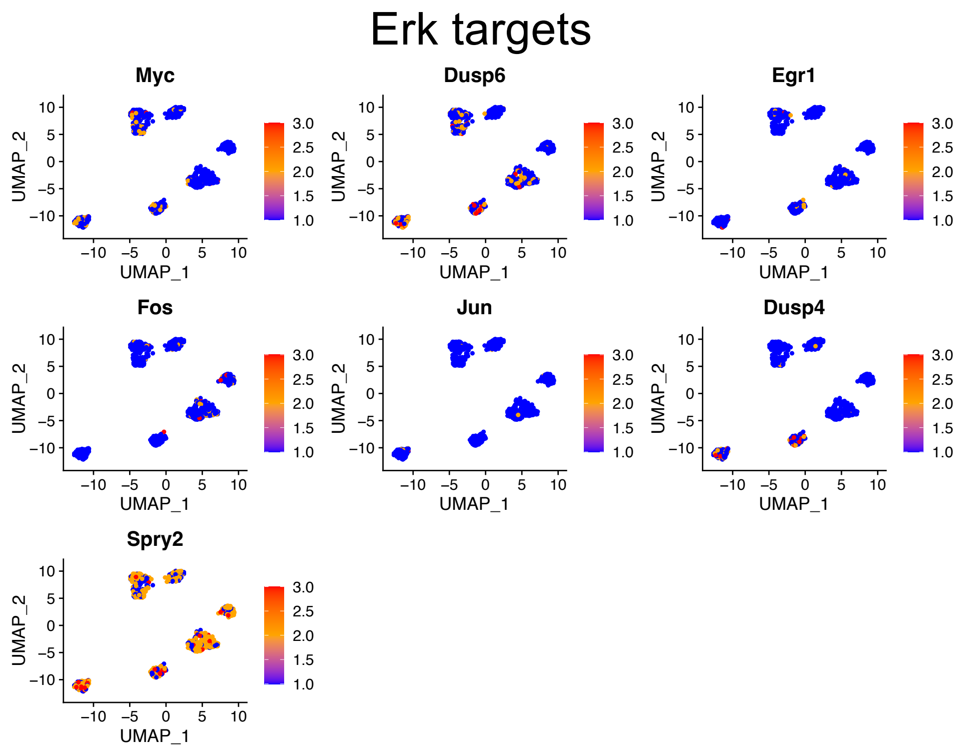

C

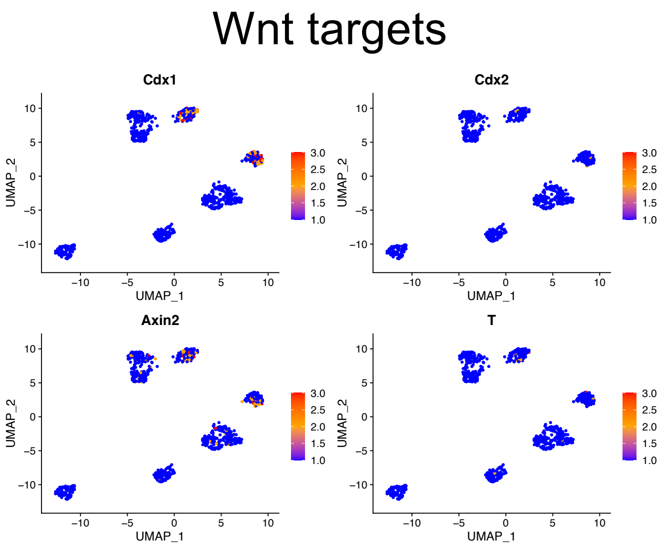

Fig. S2. Differential expression analysis within the single cell RNA-seq dataset. A. Table summarising the number of cells sequenced and the cells which passed quality control. **B., C.** Expression heatmaps superimposed upon the UMAP plot from **Figure 2B** showing the log2 of the normalised expression level of indicated genes which were identified as ERK responsive (**B**) or WNT responsive (**C**) during a 2iL withdrawal time course (Kalkan et al., 2017).

Biology Open • Supplementary information

Table S1.

Available for download at
https://journals.biologists.com/bio/article-lookup/doi/10.1242/bio.060101#supplementary-data

Table S2.

Available for download at
https://journals.biologists.com/bio/article-lookup/doi/10.1242/bio.060101#supplementary-data

Table S3.

Available for download at
https://journals.biologists.com/bio/article-lookup/doi/10.1242/bio.060101#supplementary-data

Table S4.

Available for download at
https://journals.biologists.com/bio/article-lookup/doi/10.1242/bio.060101#supplementary-data
